# Research Progress of Cell Membrane Biomimetic Nanoparticles for Tumor Therapy

**DOI:** 10.1186/s11671-022-03673-9

**Published:** 2022-03-22

**Authors:** Xuefen Zhao, Chao Yan

**Affiliations:** 1 Northern Jiangsu People’s Hospital, Yangzhou, 225001 People’s Republic of China; 2 The Affiliated Huai’an Hospital of Xuzhou Medical University and The Second People’s Hospital of Huai’an, No. 62, Huaihai Road (S.), Huai’an, 223002 People’s Republic of China

**Keywords:** Biomimetic nanoparticles, Cell membrane, Tumor therapy

## Abstract

Nanoparticles have unique properties and high design flexibility, which are thought to be safe, site-specific, and efficient drug delivery systems. However, nanoparticles as exogenous materials can provide recognition and be eliminated by the body’s immune system, which considerably restricts their applications. To overcome these drawbacks, natural cell membrane coating method has attracted great attention in the field of drug delivery systems, which can prolong nanoparticles blood circulation time and avoiding the capture as well as elimination by the body immune system. Biomimetic nanoparticles via a top-down approach can avoid the laborious group modified engineering and keep the integrity of cell membrane structure and membrane antigens, which can be endowed with unique properties, such as immune escape, longer blood circulation time, targeting delivery and controlling drugs sustain-release. At the present research, erythrocyte membrane, cancer cell membrane, platelet membrane, lymphocyte membrane and hybrid membrane have been successfully coated into the surface of nanoparticles to achieve biological camouflage. Thus, integrating various kinds of cell membranes and nanoparticles into one system, the biomimetic nanoparticles can inherit unique biofunction and drug delivery properties to exhibit tumor targeting-delivery and antitumor outcomes. In this article, we will discuss the prospects and challenges of some basic cell membrane cloaking nanoparticles as a drug delivery system for cancer therapy.

## Introduction

Nanoparticle-based drugs delivery system for disease treatment can enhance drugs accumulation at the lesion sites through retention and permeability effect, which can overcome complex biological environment to enhance therapeutic effects and avoid unnecessary side effects [1, 2]. However, considerable efforts have been devoted to nanoparticles. The exploitation of nanoparticles with optimized properties still have some challenges, such as post-nanoparticles entry to the blood circulation. This can be easily eliminated by the liver, spleen, reticuloendothelial system, and body immune system, resulting in a short half-life time, and limited nanoparticles accumulated at the lesion sites [3, 4].

Although the development of nanoengineering and modifying biomaterials on the surface of nanoparticles have achieved remarkable progress, the unanticipated material properties can negatively influence the function of nanoparticles in physiologically relevant systems [5, 6]. Thus, targeting ability, stability and biocompatibility are the three basic elements for the ideal nanoparticle. In recent years, a new type of bionic nanoparticle has been composited by the biologically derived cell membrane (versus cells and vesicles) coated nanoparticles cores, which can surpass the limit of the traditional surface modification approach [7]. Cell membrane biomimetic nanoparticles provide a top-down method to design a multifunctional drug delivery system. This is done by utilizing the complexity and versatility of the cell membrane to cloak nanoparticles and transfer the inherent characteristic of the cell membrane to the surface of the nanoparticle to execute a particular function [8].

As an effective and simple biomimetic strategy, cell membrane camouflaged nanoparticles can keep their membrane structure and antigens and realize special functions, including prolonging blood circulation time, immune escape, and specific recognition [9–11]. Moreover, different cell membranes cloaked into the surface of nanoparticles exhibits different excelling features and functions, such as platelet-membrane coated nanoparticles possessed tumor-targeting properties, leukocyte membrane cloaked nanoparticles having endothelium traversing ability, and erythrocyte membrane cloaked nanoparticles having longer blood circulation times (As Table 1 shows) [12, 13]. As the literatures reported that only various cancer cells and blood circulation cell membrane have been successfully coated into the surface of the nanoparticles platform to achieve biological camouflage at present stage [14]. After integrating cell membranes and nanoparticles into one system, the biomimetic nanoparticles can inherit unique biofunction and drug delivery properties to present excellent targeting ability and antitumor outcomes. Thus, in this article we will summarize the future perspective and challenges of some basic cell membrane cloaked nanoparticles as a drug delivery system for cancer therapy.

**Table 1:** Biomedical functional of various cell membrane

Cell membrane	Properties	Loading materials
Erythrocyte Membrane Mimicking Nanoparticles	1. Immune escape 2. Longer blood circulation	1. Glucose oxidase 2. Prodrug 3. Photosensitizer
Cancer Cell Membrane Mimicking Nanoparticles	1. Homologous targeting 2. Immune escape	1. Indocyanine green 2.Poly(lactic-co-glycolic acid) 3. MnO_2_ coated MOFs
Platelet Mimicking Nanoparticles	1. Tumor-targeting 2. Immune escape	1. Nanogel 2. Liposome
Lymphocyte Biomimetic Nanoparticles	1. Inflammation 2. Targeting ability 3. Immune induction	1. ICG-PLGA 2. DOX
Hybrid membrane Biomimetic Nanoparticles	1. Longer blood circulation 2. Immune escape 3. Homologous targeting 4. Immune induction	1. Different kinds of nanocarrier 2. Drugs

## Erythrocyte Membrane Mimicking Nanoparticles

Erythrocyte is an extremely common and abundant blood cell with longer blood circulation time and transportation of oxygen to various organs and tissues [15, 16]. The mechanism of erythrocyte membrane (EM) cloaked nanoparticles has a longer circulation time in the blood, although not very clear. The main evidence is that CD47, as an integrin-associated protein, is a self-marker of EM, which can communicate with the signal regulatory protein-alpha and can combine with its corresponding receptor of macrophages [17, 18]. Thus, EM biomimetic nanoparticles have longer blood circulation time, immune escape abilities, and can easily pass through the cardiovascular system.

**Fig. 1:**
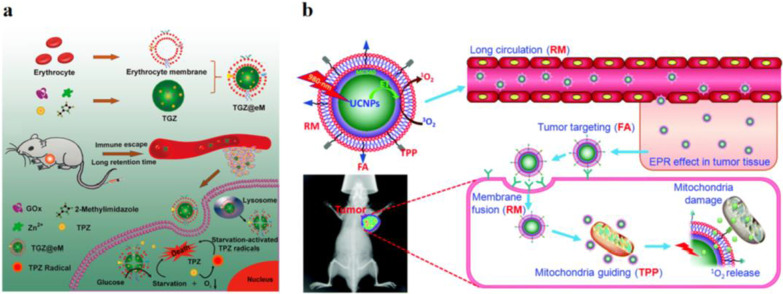
**a** Schematic Illustration of the preparation of the TGZ@eM nanoreactor and erythrocyte membrane cloaked MOF biomimetic nanoreactor starvation-activated colon cancer therapy [19]. Copyright 2018 American Chemical Society. **b** Schematic illustration of UCNP-based EM-coated dual-targeted biomimetic NPs and dual-targeted feature of the agents facilitating the uptake of the target cancer and their efficient location at mitochondria [24]. Copyright 2015 The Royal Society of Chemistry

Based on these merits, Zhang and his co-workers provided EM coated metal–organic frameworks (MOFs) based biomimetic nanoparticles loading glucose oxidase and prodrug (TGZ@eM) for starvation colon cancer therapy (Fig. 1a shows) [19]. Glucose, as the major energy source, played an essential role in providing energy for tumor metabolism. After entering the tumor areas, TGZ@eM nanoparticles displayed a burst releasing behavior of payload drugs within 2 h at pH 5.5. The releasing GOx can consume endogenous glucose and O_2_ by the enzyme-catalyzed reaction to promote tumor starves therapy. Meanwhile, the seriously hypoxic tumor microenvironment can transform prodrug into a highly cytotoxic radical to induce apoptosis of tumor cells.

Moreover, only a minor dim red fluorescence signal was detected after RAW264.7 cells were incubated with Rhm B-GOx-ZIF@eM. This result indicated that EM biomimetic nanoparticles have an immune escape ability from the body’s immune system, causing its longer blood circulation time. In vivo and in vitro tumor therapy outcomes indicated that EM biomimetic nanoparticles exhibit excellent synergistic colon cancer therapy with an accurate and facile approach.

Photodynamic therapy (PDT) has attracted great attention in disease treatment due to its noninvasive, great spatial temporal control, and avoidance of unnecessary side effects [20, 21]. Photosensitizer (PSs), light irradiation, and an abundance of oxygen are instrumental in the PDT therapeutic procedure [22]. Various methods have been exploited to optimize the properties of PSs via endowing PSs with the recognition ability or improving the singlet oxygen generation ability. Erythrocyte is the oxygen carrier in the blood, and when coated on the surface of nanoparticles can promote the permeation of ground-state molecular oxygen and the singlet oxygen compared with other coating types [23]. As Fig. 1b shows, Ding et al. developed the upconversion nanoparticle based EM coated biomimetic PDT agents and modified them with folate acid (FA) and triphenylphonium (TPP) (F/P-RM:Us/PS) on the surface for tumor therapy, which can target delivery to cancer cells and mitochondrial, respectively [24]. Comparing with Us/PS coated with EM (RM:Us/PS), the cloak effect of EM was kept at a minimal compromise once EM was modified with FA and TPP. After F/P-RM:Us/PS injected into the tumor-bear mice, the fluorescence signal was detected at 1 h later and became much stronger as time elapsed, indicating that F/P-RM:Us/PS nanoparticles have good targeting ability and can disguise nanoparticles as “self” during blood circulation. Simultaneously, F/P-RM:Us/PS biomimetic nanoparticles possess program delivery and near-infrared irradiation (NIR) -activate ability, which can effectively inhibit tumor growth and prolong the survival rate of the mice.

Thus, EM biomimetic nanoparticles can retain various membrane proteins, glycans, CD47, and acidic sialylmoieties of EM, and effectively reduce biomimetic nanoparticles' nonspecific uptake by macrophage during blood circulation [25–27]. After the surface of EM is modified with targeting groups, its immune escape ability can compromise to some extent, but the surface of targeting biomimetic nanoparticles can retain the structure and proteins integrity. This can efficiently enhance biomimetic drug delivery system targeting and reduce unnecessary side effects to normal organs. Thus, EM and nanoparticles combined to fabricate a biomimetic delivery system can enhance nanoparticles' biocompatibility, stability, and targeting ability based on the prolonged circulation time and immunity escaping ability.

## Cancer Cell Membrane Biomimetic Nanoparticles

Erythrocyte membranes undergo cloaking to prolong nanoparticles in the blood circulation time, however do not have any targeting ability against cancer cells [28, 29]. To date, some approaches have been made to improve the targeting ability by surface decoration with targeting molecules. These fabricate progress are complicated, and surface modifications can activate the body's immune system to some extent [30–32].

More evidence illustrates that cancer cells have unique targeting delivery and immune escape ability due to homologous cancer cells easy aggregation and interaction with the receptor and molecular (galectin-3, the endothelium-expressed β-galactoside-binding protein, tumor-associated Thomsen-Frieden Reich glycoantigen) on the surface of cancer cells [33, 34]. Cancer cells exhibit strong cell–cell communication and escape immune attack ability in the blood based on these merits. Thus, cancer cell membrane (CCM) biomimetic nanoparticles can select accumulation and longer retention at the tumor areas based on the homologous targeting.

**Fig. 2:**
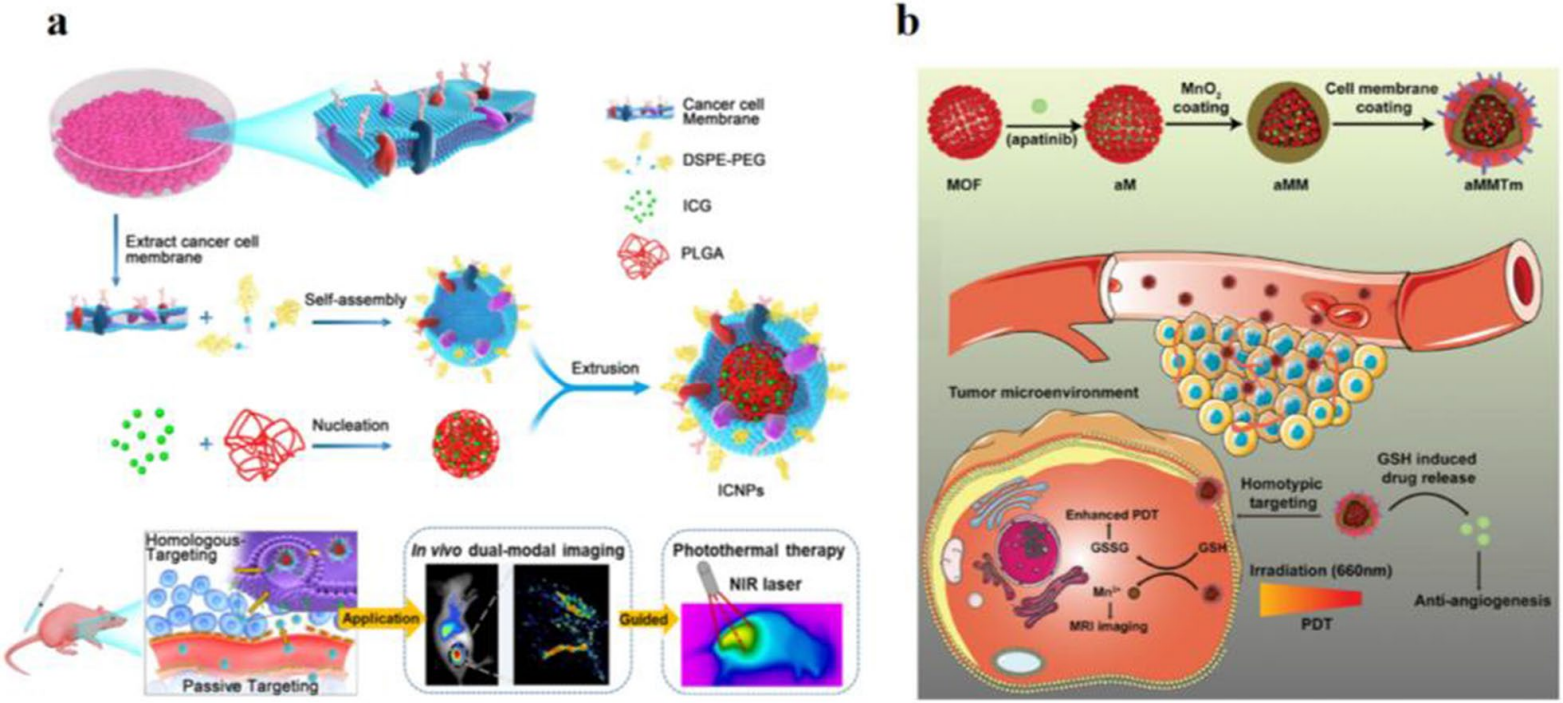
**a** Illustration of the cancer cell membrane-biomimetic ICNPs nanoparticles for targeting recognition of source cancer cell, dual-modal imaging, and photothermal therapy [35]. Copyright 2016 American Chemical Society. **b** Schematic illustration of aMMTm preparation and proposed combination therapy of PDT and antiangiogenesis [37]. Copyright 2019 WILEY–VCH Verlag GmbH & Co. KGaA, Weinheim

As Fig. 2a shows, Chen et al. fabricated a core–shell indocyanine green (ICG) loading and cancer cell membrane cloaking nanoparticle (ICNPs) for theragnostic cancer nanoplatforms [35]. MCF-7 cells were incubated with ICNPs, ICG, and INPs (ICG/poly(lactic-*co*-glycolic acid)) for 2 h. A stronger fluorescence signal was detected in the cellular cytoplasm of the ICNPs group than in the other two groups. ICNPs were injected into the tumor-carrying nude mice to investigate biodistribution. Most ICNPs were accumulated at the tumor areas via homologous targeting. Only a small amount of ICNPs was detected in the kidney and liver due to MCF-7 cell membrane can disguise ICNPs as cells to decrease kidney and liver interception. ICNPs have high spatial resolution, deep penetration, and real-time dual-modal image monitoring, completely eradicating tumors without tumor relapse upon the combination with near-infrared light irradiation. The survival rate was 100% after 18 days of therapy.

Besides, cancer cells generate high levels of glutathione (GSH), consuming ROS during PDT therapy and compromise cancer therapy outcomes [36]. Thus, ameliorating the tumor microenvironment to improve therapy efficiency should be a powerful approach for tumor therapy. As Fig. 2b shows, Min and his co-workers developed porphyrinic zirconyl-based MOFs nanoparticles, loading vascular endothelial growth factor receptor 2 inhibitor apatinib and wrapped with MnO_2_ to neutralize the intratumorally high levels of GSH. The surface was further coated with a 4T1 cancer cell membrane (aMMTm) [37]. After intravenous injection, aMMTm had longer blood half-time and accumulated at the tumor area via homotypic targeting. MnO_2_, as to reduce agent scavenger, can consume high levels of GSH at the tumor areas to enhance PDT outcomes. The reaction product Mn^2+^ can be used as a magnetic resonance imaging contrast agent to detect antiangiogenic drug delivery and distribution in vivo. The obtained multifunctional aMMTm nanosystem combined PDT can significantly enhance tumor outcomes and prolong the survival rate of 4T1 bearing mice.

The CCM biomimetic approach provides a novel strategy to achieve great progress in cancer therapy. However, the CCM biomimetic approach has some shortcomings at the early stage, including the requirements of homologous cancer cells for incubation and prolonged time and post-progressing to obtain CCM. Although CCM biomimetic nanoparticles have some drawbacks, l believe CCM biomimetic nanoparticles are a simple and efficient approach to construct an ideal drug delivery system for cancer treatment in the future.

## Platelet Mimicking Nanoparticles

Platelet originates from the cytoplasm of megakaryocytes. The surface of the platelet membrane expressed CD47, can inhibit immune elimination by the body’s immune system and prolong their circulation time in the body’s blood [38–40]. Moreover, platelet plays an important role in tumor metastasis, bacterial infection, thrombogenesis, immune escape, and other functions, due to the surface of platelet membrane having unique receptors, antigens, and proteins, such as CD59, P-selection, CD55, and glycoprotein (GP) Ib [41–45]. Thus, platelet membrane (PM) biomimetic nanoparticles can target delivery to injured vascular areas, surrounding and aggregating the tumor cells, due to the surface of PM retaining the integrity of various biomolecules. This can mediate a series of molecular interactions and promote their affinity between tumor cells and platelet membranes [46, 47].

**Fig. 3:**
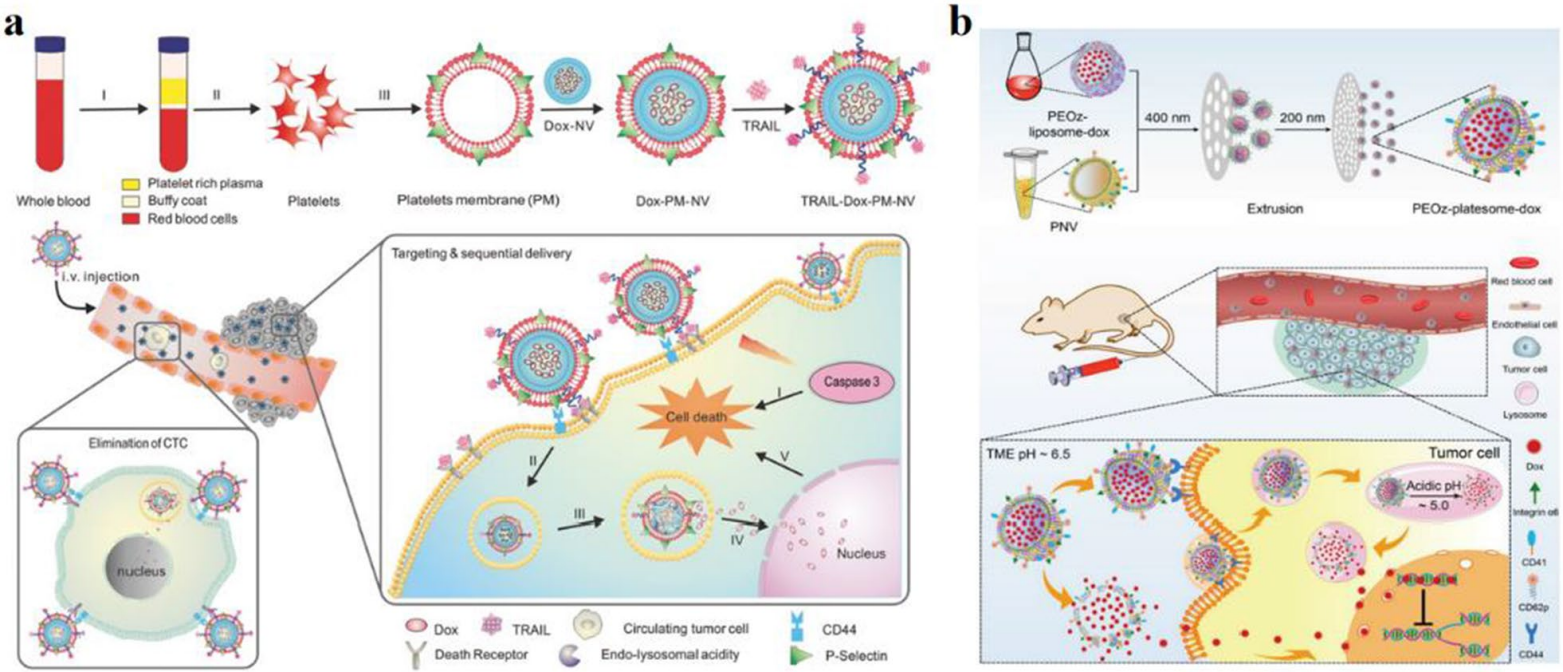
**a** Schematic design of drug-loaded PM-NV for targeting and sequential drug delivery [48]. Copyright 2016 Adv. Mater. **b** Schematic design of engineering biomimetic nanocarrier for pH-responsive drug delivery and enhanced anti-tumor activity [49]. Copyright 2019 WILEY–VCH Verlag GmbH & Co. KGaA, Weinheim

As Fig. 3a shows, Hu et al. developed PM camouflaged core–shell nano vehicle (PM-NV), the nanogel core used to load drugs. The surface of PM was further decorated with protein drugs, which can achieve targeting delivery and site-specific releasing behavior [48]. Tumor necnosis factor-related apoptosis-inducing ligand (TRAIL) and doxorubicin (DOX) as the most important extracellular activators of apoptosis, were simultaneously incorporated into PM-NV (TRAIL-DOX-PM-NV) for tumor therapy. TRAIL-DOX-PM-NV can be endocytosed and digested after incubating with tumor cells, promoting DOX accumulation at the nuclei and improve tumor cell apoptosis. When administered into the tumor mice model, TRAIL-DOX-PM-NV has blood stability, targeting delivery, and immune escape ability, which can aggregate the tumor cells to inhibit cancer cell metastatic and enhance tumor therapy.

After the platelet membrane is fused with other membranes, the obtained products inherit the function of the platelet membrane and retain the advantages of other membranes. As Fig. 3b shows, Liu and his co-workers designed a novel platelet membrane-lipid hybrid biomimetic pH-responsive nanoparticles (PEOz-liposome-DOX) to target delivery and accelerate drug release at tumor acidic environment [49]. During platelet membranes and the liposome fusion process, pH-sensitive lipid DSPE (1,2-dioleoyl-sn-glycero-3-phosphoethaNolamine)-PEOz and DOX were simultaneously incorporated into the hybrid membrane carrier, accelerating drug-release at acidic conditions. In mouse tumor models, PEOz-liposome-DOX nanoplatform significantly exhibited tumor accumulation capacity, longer half-time, and excellent anti-tumor effects compared to traditional nanoparticles without pH-responsive or traditional liposomes with pH-sensitive behavior.

Platelet membrane biomimetic camouflage nanoparticles have stealthy and biofriendly behavior in the blood circulation, reducing cell uptake by macrophage cells, selective adhesion to damage vasculatures, and improved nanoparticles adherence to cancer cells or pathogens to improve therapy effect.

## Lymphocyte Biomimetic Nanoparticles

Lymphocytes, including T cells, B cells, and NK cells as the typical immune cells, play a core role in the intrinsic immune system against pathogen infection and tumor progression [49]. The number of lymphocytes increases rapidly after being activated by various diseases, and have far longer half-time’s in the blood circulation [50]. However, the lymphocyte surface has some specific immune recognition proteins that can specifically recognize with correlated molecules on the surface of cancer cells and unique site-specific targeting ability [51]. Immune cells have high cancer affinity capacity, so their membrane can be an ideal drug carrier for tumor targeting delivery.

**Fig. 4:**
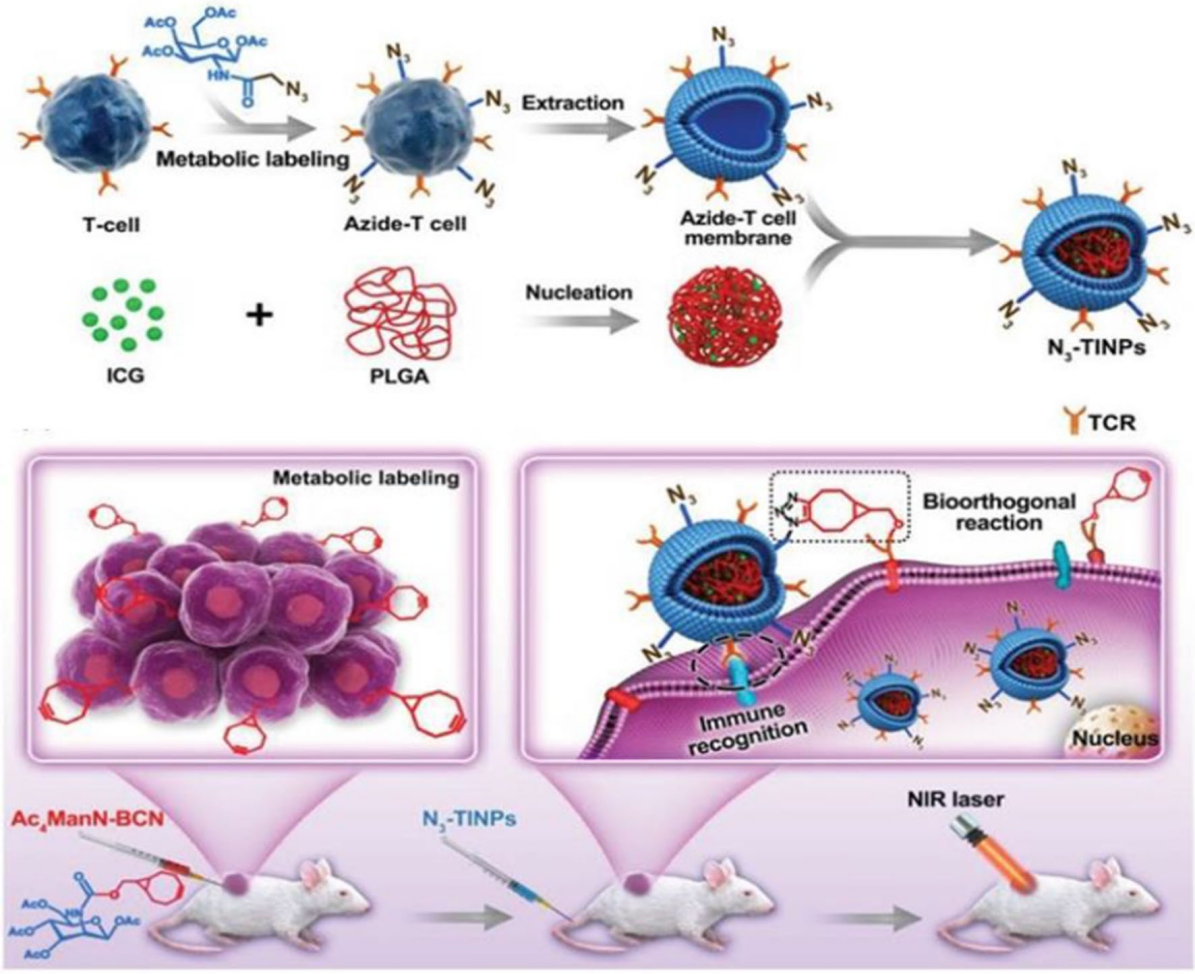
Schematic illustration of N_3_-labeled T cell membrane-biomimetic nanoparticles with a dual-targeting mechanism for highly efficient photothermal therapy [51]. Copyright 2019 WILEY–VCH Verlag GmbH & Co. KGaA, Weinheim

The inter-and intra-heterogeneity of tumor tissues result in single targeting ability was unsatisfactory, hence the dual-targeting approach is a promising strategy to improve nanoparticles accumulation at the lesion sites. Han et al. designed an azide (N_3_) labeled T-cell membrane mimicked photosensitizer to increase targeting delivery and enhance photothermal therapy outcomes [52]. As Fig. 4 shows, N_3_ groups were modified into the surface of T cell via glycometabolism with the azido sugar, a novel bicyclononyne (BCN) modified unnatural sugar (Ac_4_ManN-BCN). ICG loading into poly (lactic-*co-*glycolic acid) to form ICG-PLGA core, and then N_3_-labeled T cell membrane was coated into the ICG-PLGA core to form N_3_-TINPs. After N_3_-TINPs entered into the tumor-bearing mice, N_3_-TINPs detected the strongest fluorescence intensity than TINPs (T cell membrane coated ICG-PLGA core) and ICG treatment, which indicated N_3_-TINPs possessed excellent dual-targeting capacity and can effectively accumulate at the tumor areas. Additionally, N_3_-TINPs possess outstanding distinct tumors to inhibit effects with no relapse during 16 days of treatment and have excellent biosafety. The fluorescence signal of ICG was detected in fecal, which indicated that N_3_-TINPs were mainly metabolized from the liver into the intestine and excreted by fecal. This dual-targeting biomimetic nanoparticle has high targeting ability, and tumor eradicating outcomes.

There is approximately 5–20% of natural killer (NK) cells in the peripheral blood mononuclear and tissues including peritoneal cavity, placenta, and liver, which play a predominant member in the innate immune system [53–55]. NK cells fight against cancer cells, microbial infections and allogeneic cells with stress markers via a host defense by immunosurveillance of the surface of cells abnormalities in cell stress markers and major histocompatibility class I markers in the surface of NK cells [56, 57]. The mechanism of NK cells killed cancer cells involves releasing correlation proteolytic enzyme and membrane disruption protein. The results illustrated that cancer apoptosis relies on the overexpression of NK cells receptor ligands in cancer cells (NKG2-D) [54]. Unlike other immune cells, various mechanisms of NK cells can directly be targeting cancer cells, including inhibitory and activating correlation receptors on NK cell surface, interferon-γ effector function, death receptor-induced apoptosis, and perforin/granzyme mediated cell cytotoxicity [58].

**Fig. 5:**
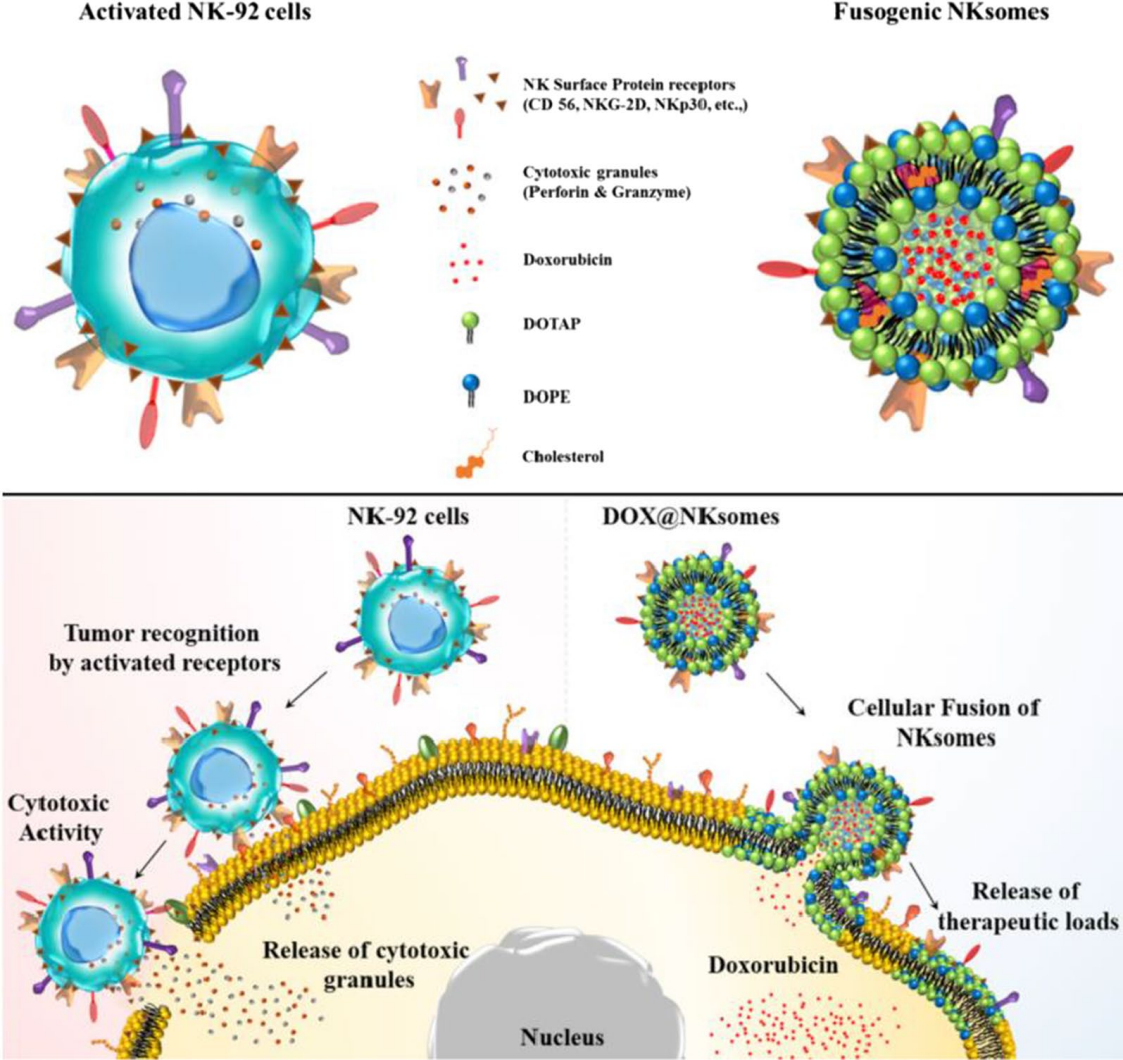
Schematic illustration of activated NK cells (NK-92 cells) and NK-92 cell membrane-derived fusogenic liposomes (NKsomes) for targeted tumor therapy [63]. Copyright 2018 Elsevier Ltd

NK cells and their products were utilized for immunotherapy just in Phase I clinical trials. However, NK cells membrane mimicking nanoparticles exhibited outstanding therapeutic effect in the model of mouse tumor xenografts [59–62]. Among various NK cell lines, NK-92 cells lack the inhibitory receptors of NK cells, and ease expansion for immunotherapy. The surface presented CD56 receptors, and activated receptors for cytolytic function, has achieved excellent anti-tumor activation in a mice xenograft model of human leukemia and melanoma [63, 64]. As Fig. 5 shows, Arunkumar Pitchaimani et al. developed NK cells membrane biomimetic nanocarrier (NKsomes) via activated NK-92 cell membrane and extruded with the fusogenic liposome, which have longer circulation time, is nonimmunogenic, and higher targeting delivery ability [63]. After DOX was loaded into the core of NKsomes (DOX@NKsomes), it exhibited high stability under normal physiological condition up to two weeks. After entering the tumor acidic environment, DOX@NKsomes core showed nearly 75% in drug-release behavior. In contrast, bare liposomes core showed about 88% drug-release, which indicates that DOX@NKsomes has sustained and environment responsive drug release behaviors. DOX@NKsomes' targeting ability, superior toxicity, longer half-time, tumor accumulation efficacy, and homing ability make them outstanding tumor therapy effects with longer mice survival rates than other treatment methods.

Above all, tumor homing ability of lymphocyte for tumor targeting therapy through utilizing the properties of the different immune cells' membrane, opens a new window to design various kinds of biomimetic nanoparticles based on patient's demand.

## Prospects and Challenges

Nanoparticles biomimetic approach has been well-designed for drug delivery system with immune escaping, active targeting, longer blood circulation time, excellent tumor therapy outcomes, and minimal systemic toxicity compared with traditional drug delivery systems [65]. However, the application and investigation of biomimetic nanoparticles are still at an infant stage. There exist various challenges and problems that need to be solved, including the source of the cell membrane, the fabrication process of biomimetic nanoparticles, and the safety, biocompatibility, targeting ability of these biomimetic nanoparticles in tumor therapy.

Different cell membranes have different sources, such as erythrocytes and platelets, which come from the body's common and abundant blood cells where they can be easily obtained [66]. Tumor cell membrane and immune cell membranes however, require tedious fabrication process, including large samples, cultivation, and amplification in vitro [67]. Biomimetic nanoparticles as foreign materials can induce a robust immune response after injection into the body. Therefore, design and construction of homologous cell membrane-based biomimetic nanoparticles origin from the patients’ lesion areas should be considered.

Uneven or incomplete coverage of nanoparticles may easily induce the body’s immune response to eliminate the biomimetic nanoparticles system. Thus, keeping the integrity of the cell membrane structure is an important problem to be considered during the fusion and extraction process [68]. At present, repeating the freeze–thaw process and the hypotonic treatment is the most common extraction approach to maintain the cell membrane's functions and integrity [69]. However, these current processes are still stagnant at the early research stage, which require more time and procedures to optimize until they meet the clinical applications.

Safety and biocompatibility are the most important factors before approval for clinical application. Currently, cell membrane biomimetic technology exhibits good biocompatibility and targeting ability compared to other traditional modified methods. However, these trials are still staying at a simple and primary mice experiment, which require more in vivo detail experiments and information to illustrate.

Above all, cell membrane biomimetic nanoparticles exhibit superior tumor therapy effects after being combined with the advantages of cell membrane and nanoparticles. After cell membranes are modified with targeting molecules and integrated with nanoparticles or other therapeutic agents, biomimetic nanoparticles can realize excellent tumor therapy outcomes. However, these works are still at the early stage, which require repeatability experimentation before clinical use.

## Conclusion

In summary, the cell membrane biomimetic approach has made a great contribution to tumor therapy. This article summarizes some types of cell membrane (erythrocyte membrane, cancer cell membrane, platelet membrane, and leukomonocyte membrane) biomimetic nanoparticles to endow them with longer blood circulation time, immune escape, and tumor targeting ability to realize favorable anti-tumor effects. However, the clinical application of biomimetic nanoparticles encounters many challenges to resolve, including complex fabrication process, unsuitable large-scale production, low yields, and difficult preservation. Overall, targeting delivery, excellent anti-tumor properties, prolonged circulation time, minimal side effects, and positive economic effects should be the elemental factors of the cell membrane biomimetic approach for translating into clinical utilization.

## Data Availability

Not applicable.

## Abbreviations

EM: Erythrocyte membrane
MOFs: Metal–organic frameworks
GOx: Glucose oxidase
TGZ@eM: EM coated MOFs based biomimetic nanoparticles loading GOx and prodrug
PDT: Photodynamic therapy
PSs: Photosensitizer
FA: Folate acid
TPP: Triphenylphonium
CCM: Cancer cell membrane
ICG: Indocyanine green
ICNPs: ICG loading and cancer cell membrane cloaking nanoparticle
GSH: Glutathione
ROS: Oxygen reactive species
NIR: Near-infrared irradiation
aMMTm: A 4T1 cancer cell membrane
GP: Glycoprotein
PM: Platelet membrane
PM-NV: PM camouflaged core–shell nano vehicle
DOX: Doxorubicin
N_3_: Azide
BCN: Bicyclononyne
Ac_4_ManN-BCN: BCN modified unnatural sugar
lactic-*co-*glycolic acid: ICG loading into poly
NK: Natural killer
NKG2-D: NK cells receptor ligands in cancer cells
NKsomes: NK cells membrane biomimetic nanocarrier
DOX@NKsomes: DOX was loaded into the core of NKsomes
